# End Criteria for Reaching Maximal Oxygen Uptake Must Be Strict and Adjusted to Sex and Age: A Cross-Sectional Study

**DOI:** 10.1371/journal.pone.0085276

**Published:** 2014-01-14

**Authors:** Elisabeth Edvardsen, Erlend Hem, Sigmund A. Anderssen

**Affiliations:** 1 Norwegian School of Sport Sciences, Department of Sports Medicine, Oslo, Norway; 2 Oslo University Hospital, Ullevål, Department of Pulmonary Medicine, Oslo, Norway; Oregon Health & Science University, United States of America

## Abstract

**Objective:**

To describe different end criteria for reaching maximal oxygen uptake (VO_2max_) during a continuous graded exercise test on the treadmill, and to explore the manner by which different end criteria have an impact on the magnitude of the VO_2max_ result.

**Methods:**

A sample of 861 individuals (390 women) aged 20–85 years performed an exercise test on a treadmill until exhaustion. Gas exchange, heart rate, blood lactate concentration and Borg Scale_6–20_ rating were measured, and the impact of different end criteria on VO_2max_ was studied;VO_2_ leveling off, maximal heart rate (HR_max_), different levels of respiratory exchange ratio (RER), and postexercise blood lactate concentration.

**Results:**

Eight hundred and four healthy participants (93%) fulfilled the exercise test until voluntary exhaustion. There were no sex-related differences in HR_max_, RER, or Borg Scale rating, whereas blood lactate concentration was 18% lower in women (*P*<0.001). Forty-two percent of the participants achieved a plateau in VO_2_; these individuals had 5% higher ventilation (*P = *0.033), 4% higher RER (*P*<0.001), and 5% higher blood lactate concentration (*P = *0.047) compared with participants who did not reach a VO_2_ plateau. When using RER ≥1.15 or blood lactate concentration ≥8.0 mmol•L^–1^, VO_2max_ was 4% (*P* = 0.012) and 10% greater (*P*<0.001), respectively. A blood lactate concentration ≥8.0 mmol•L^–1^ excluded 63% of the participants in the 50–85-year-old cohort.

**Conclusions:**

A range of typical end criteria are presented in a random sample of subjects aged 20–85 years. The choice of end criteria will have an impact on the number of the participants as well as the VO_2max_ outcome. Suggestions for new recommendations are given.

## Introduction

The measurement of maximal oxygen uptake (VO_2max_) has been available for more than half a century and provides useful information about an individual's maximal cardiorespiratory fitness and level of physical performance. During the exercise test, the technicians' skills and the subjects' motivation and effort are important requirements to ensure valid and reliable results when comparing groups in large epidemiological surveys, as well as for the accurate interpretation of a maximal test for both athletes and patients.

The classical plateau described by Taylor and coworkers is recognized as the gold standard to determine a true VO_2max_ [1]. However, this criterion is not straight forward to use in practical settings [2]. Therefore, a large variety of other end criteria have been used, such as an elevated respiratory exchange ratio (RER) ≥1.0 [3]–[5], 1.10 [6], [7], or 1.15 [8], [9], the achievement of a certain percentage of the age-adjusted estimate of HR_max_ [7], [10], [11], high postexercise blood lactate levels (≥8 mmol•L^–1^) [8], [12], the subject's rating of perceived exertion (Borg Scale rating or Visual Analog Scale) [13], or a combination of the above mentioned variables [14]. Thus, there is currently no consensus regarding the assessment of maximal effort during a continuous graded exercise test on the treadmill – especially among women and the elderly – and the knowledge about how different end criteria variables are affected by gender and aging is scarce. Furthermore, the original recommendations are often based on older studies that used measurement equipment and test protocols that are different from those used today [1], [15], and the number of participants was low [7], [9], [16], [17] or consisted of athletes or children and adolescents [7], [15], [18]–[20]. Therefore, the purpose of this study was to describe the different end criteria that are used often for reaching VO_2max_ during a maximal progressive graded exercise test on the treadmill in a healthy sample of 20–85-year-old men and women, and to explore if the choice of end criteria has an impact on the VO_2max_ value.

## Materials and Methods

### Ethics Statement

The study was approved by the Regional Committee for Medical Ethics (REK South-Eastern Norway B, S-08046b), the Norwegian Social Science Data Services AS, and the Norwegian Tax Department. All individuals signed written informed consent forms before participating.

### Study Design

This study was a cross-sectional multicenter study involving nine test centers from all regions of Norway. The participants were healthy men and women aged 20–85 years who participated in the population-based KAN study carried out in 2008/2009 [21]. The only inclusion criterion was age-related, and 1,930 of the subjects were randomly invited to participate in a sub study during 2009–2010, including a cardiopulmonary exercise test (CPET) on a treadmill [14]. Finally, a total of 904 men and women met at the laboratory and 804 completed CPET to exhaustion.

### Exercise Test

Height and body weight were measured to the nearest 0.5 cm and 0.1 kg, respectively, with participant's wearing no shoes and light clothes. The exercise test was performed during daytime by walking and running on a treadmill using a modified Balke protocol [22]. Four minutes of warm-up were performed with the treadmill speed set at 4.8 km·h^–1^ and inclination set at 4%. For participants who were older than 55 years or were obese, the speed was set at 3.8 km·h^–1^. The inclination was then increased each 60 s by 2%, up to a 20% inclination. If the participant was still able to continue, the speed was further increased by 0.5 km·h^–1^ until exhaustion. Gas exchange and ventilatory variables were measured continuously as the subjects breathed into a Hans Rudolph two-way breathing mask (2700 series; Hans Rudolph Inc., Shawnee, KS, USA). During the last part of the test, the subject's effort was largely encouraged by the technician until voluntary termination. The rating of perceived exertion was obtained using the Borg Scale_6–20_ [23]. A capillary blood sample was taken 60 s after termination of the exercise test and analyzed for blood lactate concentration using hemolyzed blood (Lactate Pro; KDK Corporation, Kyoto, Japan; or ABL 800; Radiometer Medical, Copenhagen, Denmark).

The gas analyzers used were daily volume- and gas calibrated corrected for barometric pressure, temperature and humidity. A detailed descriptions regarding measurement accuracy between gas analyzers is given elsewhere [14]. The gas-exchange variables were reported as 30 s averages. HR was recorded each minute using a Polar Sports Watch (Kempele, Finland) or 12-lead ECG. The highest VO_2max_ during 30 s stage was used, and the highest RER measured before or corresponding to the last 30 s stage was reported. A plateau in VO_2_ was defined as any two 30-sec VO_2_ values in which the second was not higher than the first, provided increase in ventilation at maximal effort. Participants who did not exhibit an increase in ventilation despite achievement of a plateau were not accepted. This to ensure that the subject had reached the respiratory compensation point caused by metabolic acidosis.

The different end criteria used to study the impact on VO_2max_ were VO_2_ leveling off, RER_max_ ≥1.0, 1.10, and 1.15, blood lactate concentration ≥6.0 and 8.0 mmol•L^–1^, Borg Scale_6–20_ rating, and HR_max_ ≥95% of the age-predicted HR_max_ (220– age) compared with symptom-limiting termination of the test.

### Statistical Analysis

Demographic data were presented as mean values ± standard derivation (SD), and cross-sectional data were reported according to age and sex and grouped into 15-year cohorts. Analysis of variance (ANOVA) was used to evaluate differences in the end-criteria variables between age groups. A test of trend was performed with x values equal to the average within each age category. The effects of the end criteria on VO_2max_ were tested using Student's *t* test. Correlations between the commonly accepted end criteria were assessed using Pearson's correlation coefficient (r). Statistical tests were conducted using SPSS version 18.0 (SPSS, Chicago, Illinois, USA). *P* values of ≤0.05 were considered statistically significant.

The new recommendations for maximal effort are based on mean values for postexercise blood lactate concentration and RER –1 SD, which included 84% of the participants. To simplify, the blood lactate recommendations are reported to the nearest 0.5 mmol•L^–1^.

## Results

This study examined 861 subjects during exercise testing on a treadmill. Thirty subjects ended the study prematurely or were excluded because of medical considerations, and 27 participants were not able to perform the test to voluntary exhaustion. The participants' characteristics according to 15-year cohorts are shown in Table 1.

**Table 1:** Baseline characteristics of the participants by 15-year age cohort for female and male (SD).

	Age (year)			
Female	20–34	35–49	50–64	65–85
Number of subjects	62	135	121	71
Age (year)	28.5 (4.2)	42.7 (4.5)	57.6 (4.5)	72.8 (5.4)
Height (cm)	168.4 (7.6)	167.5 (5.7)	166.0 (5.4)	162.0 (6.1)
Weight (kg)	68.0 (10.1)	70.7 (14.4)	70.3 (12.0)	66.7 (9.0)
BMI	24.0 (3.7)	25.1 (4.6)	25.5 (4.1)	25.4 (3.4)
**Male**				
Number of subjects	72	135	138	70
Age (year)	29.3 (3.7)	42.6 (4.0)	57.6 (4.3)	71.0 (4.9)
Height (cm)	181.7 (5.6)	179.6 (6.5)	179.2 (6.9)	176.3 (6.6)
Weight (kg)	82.7 (11.4)	84.8 (12.7)	86.5 (11.2)	81.1 (11.0)
BMI	25.1 (3.5)	26.3 (3.6)	26.9 (3.0)	26.1 (2.9)

BMI  =  Body Mass Index.

### Maximal Exercise

The main subjective reason for stopping exercise was dyspnea in women (54%) and muscular fatigue in men (38%). General fatigue was reported in 28% of the subjects. There was no age-related influence on the reason for ending the test. The maximum end criteria variables are given in Table 2.

**Table 2:** End criteria variables during maximal exercise by 15-year age cohort for female and male (SD).

	Age (year)			
Female	20–34	35–49	50–64	65–85
VO_2_ levelling off (%)	40	39	46	37
Heart rate (beat·min^−1^)	188.1 (7.5)a	180.4 (10.4)b	170.6 (10.3)c	157.7 (15.5)
RER (VCO_2_·VO_2_^−1^)	1.21 (0.10)c	1.20 (0.11)	1.17 (0.11)	1.13 (0.12)
Blood lactate concentration (mmol·L^−1^)	9.9 (2.5)b	9.2 (2.5)b	7.4 (2.6)c	6.1 (2.5)
BORG scale_6–20_	17.8 (1.1)	17.6 (1.3)	17.5 (1.2)	17.2 (1.2)
**Male**				
VO_2_ levelling off (%)	33	46	46	36
Heart rate (beat·min^−1^)	1.93.1 (8.2)a	183.1 (11.8)b	170.1 (14.0)c	154.3 (14.4)
RER (VCO_2_·VO_2_^−1^)	1.23 (0.1)b	1.22 (0.1)c	1.18 (0.1)c	1.11 (0.1)
Blood lactate concentration (mmol·L^−1^)	12.1 (2.6)b	11.9 (2.8)b	8.8 (2.8)c	6.8 (2.6)
BORG scale_6–20_	17.9 (1.3)	17.7 (1.2)	17.4 (1.3)	17.2 (1.4)

RER  =  Respiratory Exchange Ratio; VO_2_ =  Oxygen uptake; VCO_2_ =  Carbon dioxide output; a =  the participants' end criterion was significant higher than the other age cohorts; b =  the participants end criterion was significant higher than the participants older than 49 years; c =  the participants' end criterion was significant higher than participants older than 64 years.

Forty-one percent of the women and 42% of the men achieved a plateau in VO_2_ at the end of the test. Those who achieved a plateau (n = 335) had a 4% higher RER (*P*<0.001) and a 5% higher postexercise blood lactate concentration (*P = *0.047) compared with those who did not reach a plateau; however, there was no difference in HR_max_ (*P = *0.09), Borg Scale rating (*P = *0.36), and VO_2max_ (*P = *0.181) between these two groups. Of the subjects who attained a plateau in VO_2_, 38%, 25%, and 9% did not achieve the commonly accepted end criteria of blood lactate concentration ≥8.0 mmol•L^–1^, RER ≥1.15, and ≥95% of the HR_max_ predicted, respectively.

The RER was 1.21 (±0.11) in the 20–49-year-old group of participants, and decreased to 1.12 (±0.12) in the oldest age group (*P*<0.001). There were no sex-related differences (*P = *0.09), and the association with age was weak (r = –0.304, *P*<0.001). In addition, there was no association between VO_2max_ in % predicted (r = –0.009, *P* = 0.790). Ninety-five percent, 80%, and 65% of the subjects reached an RER of 1.00, 1.10, and 1.15, respectively.

The postexercise blood lactate concentration was 12.0 (±2.7) mmol·L^–1^ in 20–49-year-old men and subsequently decreased linearly to 6.8 (±2.6) mmol·L^–1^ in the oldest age group (*P*<0.001). Women had an 18% lower (*P*<0.001) blood lactate level than men; however, there was no significant difference between sexes after age 65. A blood lactate concentration ≥8.0 mmol•L^–1^ was strongly age dependent, excluding 63% of the participants in the 50–85-year-old cohort. Among the subjects who had a lactate concentration ≥8.0 mmol•L^–1^, 18% and 17% failed to achieve an RER ≥1.15 and a Borg Scale rating ≥17, respectively. The strongest association with blood lactate concentration was observed for HR_max_ (r = 0.587, *P*<0.001) and RER (r = 0.540, *P*<0.001). The association between blood lactate concentration and VO_2max_ in % predicted was low (0.226, *P*<0.001).

The Borg Scale_6–20_ rating was 17.6 (±1.3), with no differences according to sex or age. Among those who did not fulfill the Borg criterion (Borg Scale rating<17), the blood lactate concentration and RER were 18 mmol•L^–1^ (*P*<0.001) and 5% (*P*<0.001) lower, respectively, compared with those who did. There was no association between Borg Scale_6–20_ and VO_2max_ in % predicted (0.053, *P* = 0.151).

The HR_max_ was191 (±8.25) beat·min^–1^ in the 20–34-year-old cohort, with no sex-related differences (*P = *0.311), and declined with age in both sexes by about 8.8 beat·min^–1^ (*P*<0.001) per 15-year cohort (r = –0.711, *P*<0.001). Nine subjects (1%) did not reach 85% of the HR predicted, and 79 subjects (10%) did not reach 95% of the HR predicted.

### End Criteria and Impact on Oxygen Uptake

**Figure 1:**
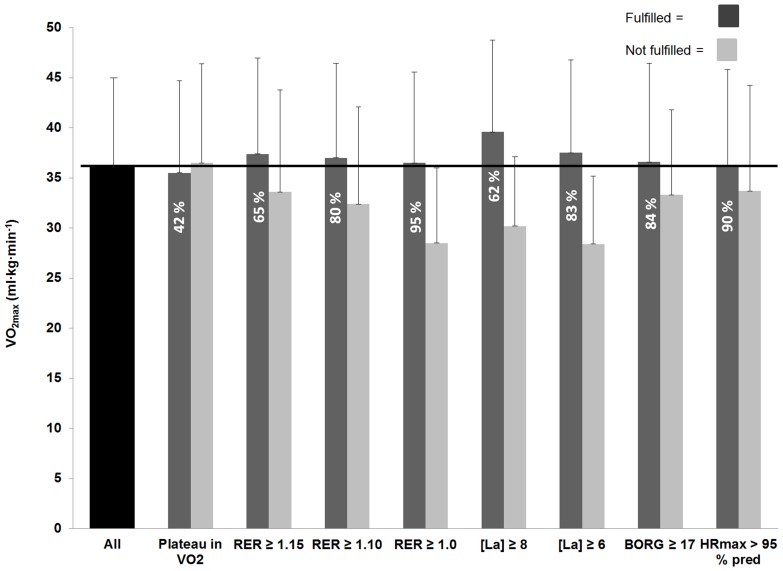
Maximal oxygen uptake (VO_2max_) using different end criteria (dark grey) compared to volitional fatigue (all) (mean ± SD). The light grey bars show VO_2max_ in those subjects who did not fulfilled the end criterion. The % of participants who fulfilled the criterion is reported on each bar.

The dark grey bars in Figure 1 shows VO_2max_ using different end criteria compared to voluntary exhaustion. When using RER ≥1.15, the VO_2max_ was 4% greater (*P* = 0.012) compared to subjects who did not reach the same criterion. Furthermore, RER ≥1.15 excluded 281 subjects (35%) from the population. After age adjustment, there was no change in VO_2max_ between the different method (*P* = 0.923). Correspondingly, when using only a blood lactate concentration ≥6.0 or ≥8.0 mmol•L^–1^, the VO_2max_ was 4% (*P* = 0.004) and 10% (*P*≤0.001) greater. The difference was highest after 50 years of age (8.5%).

The difference between the dark grey and light grey bars in Figure 1 shows the difference in VO_2max_ between the subjects who fulfilled and those who did not fulfill the different end criteria. The largest difference in VO_2max_ was observed between individuals who fulfilled and those who did not fulfill the blood lactate concentration criterion and RER ≥1.0.

## Discussion

The purpose of this study was to describe different end criteria for reaching VO_2max_ during a progressive maximal treadmill test in a healthy sample of 20–85-year-old men and women, and to explore if the choice of end criteria had an impact on the VO_2max_ value. The major findings were that the postexercise blood lactate concentration and RER decreased with age, despite the fact that the subjective ratings of exertion related to age remained unchanged. Furthermore, choosing a blood lactate concentration ≥8.0 and ≥6 mmol•L^–1^ and/or RER ≥1.15 yielded a higher VO_2max_, but excluded a significant number of participants from the analysis.

### End Criteria Variables for Maximal Oxygen Uptake

The classical criterion for VO_2max_ is achievement of a plateau in VO_2_ despite an increase in work rate. A RER above a certain level, a high level of blood lactic acid, and age-adjusted estimates of HR_max_ are also used, especially in subjects who failed to achieve a plateau [8]. The higher HR_max_ achieved in each age group compared with other similar studies [6], [19], [24] allows us to state the high validity of our data. It also reflects differences between studies in the degree of motivating the subjects to exhaustion, which underline the importance of using equal end-criteria in large epidemiological studies. There was, however, a substantial range of maximal values for each of the reported end variables according to age and sex (blood lactate concentration, 1.2–18 mmol•L^–1^; RER, 0.85–1.57; HR_max_, 75–137% predicted based on 220-age), which complicates the interpretation of the results and, thus, may be of major concern when choosing optimal criteria during exercise testing.

#### VO_2_ Plateau

A plateau in VO_2_ was found in 42% of the subjects and was defined as a VO_2_ leveling off, despite a rise in ventilation, which is in line with findings from other investigations [19], [20]. Our definition differs from the classical definition of a plateau described by Taylor and co-workers [1]. Taylor performed several systematic “steady state” tests over 3–5 days using Douglas bags, and found that the increase in VO_2_ during the treadmill protocol from day to day was approximately 4.2±1.1 mL•kg^–1^•min^–1^. Based on this observation, those authors claimed that an increase of less than 2 SD of the expected rise in VO_2_ satisfies a plateau, representing less than 2.1 mL•kg^–1^•min^–1^ to the next level, or less than 150 mL•min^–1^ if the participant's body mass was 72 kg [1].

**Figure 2:**
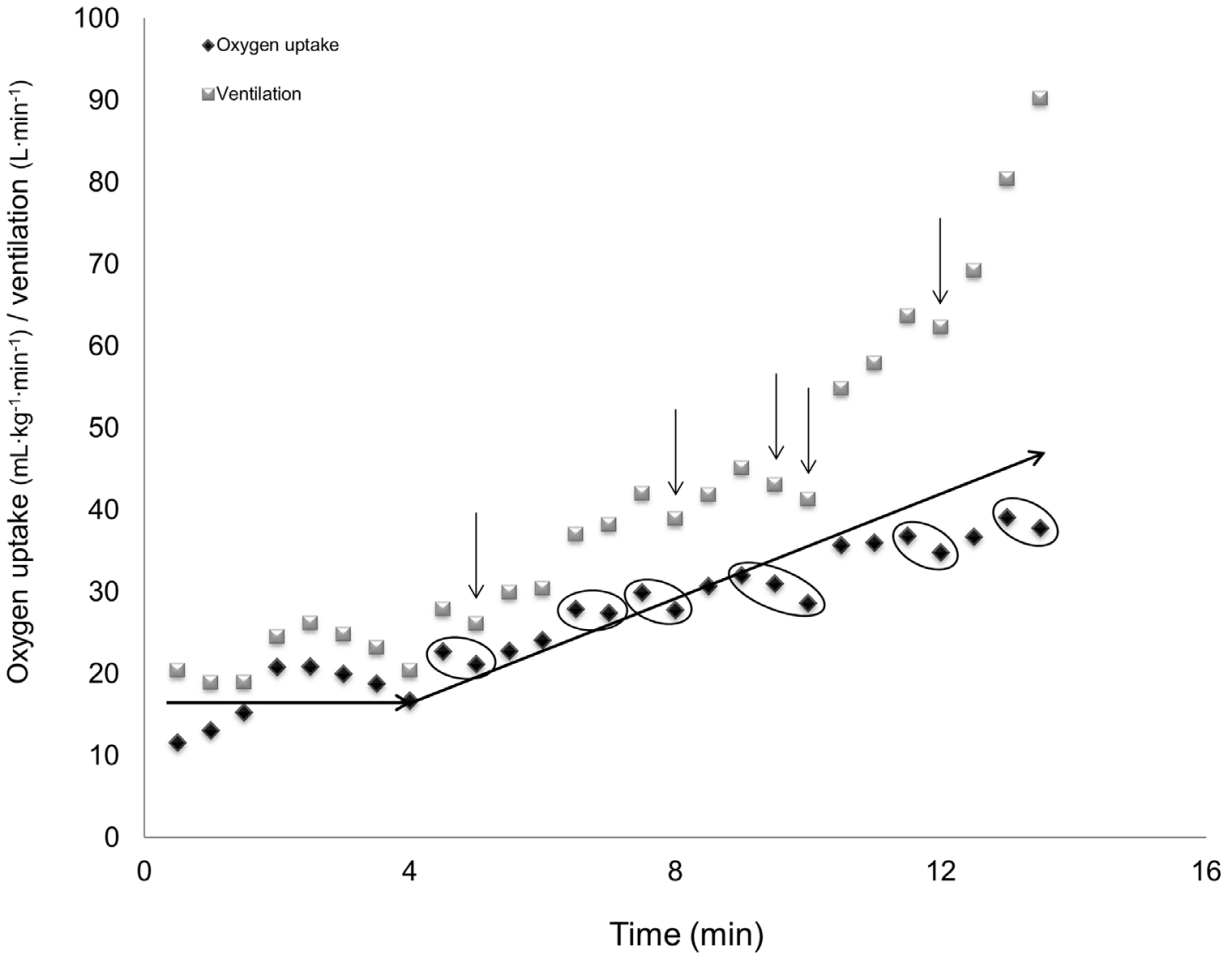
Oxygen uptake (mL•kg^−1^•min^−1^) and minute ventilation (L•min^−1^) from table 3 plotted against time (min). The arrows' pointing downward indicates a drop in ventilation followed by a levelling off in VO_2_ marked by a circle. The right pointing arrow indicates the expecting increase in VO_2_.

Despite that the Taylor's method is considered the gold standard for defining VO_2max_, there are several reasons why we did not chose this method during the continuous graded exercise protocol. First, our protocol included a much smaller increase in workload. A smaller increase in workload may lead to measurements that exhibit more fluctuation regarding VO_2_ between each sampling. As outlined in Table 3, a continuous graded protocol may lead to the achievement of several VO_2_ plateaus during the test, also above RER  = 1.15, which may preclude the recording of a valid VO_2max_. Second, the body mass of many of the participants in the current study differed substantially from 72 kg, which hampers the comparison between studies. Third, the use of minute ventilation instead of workload was chosen to ensure that the subjects had reached their respiratory compensation point at the end of the test, also illustrated in Figure 2. The respiratory compensation point reflects the final phase of exercise, at which hyperventilation occurs to decrease the arterial pCO_2_ resulting from metabolic acidosis [25]. In addition, the measurement of ventilation is online at any time, following simultaneously the subject's breathing pattern, while expiratory gases will be delayed to a greater or lesser extent depending on the size of the ventilation. Thus, if ventilation increases and oxygen uptake is constant during increased workload, it is reasonable to assume that the gas exchange has reached its maximum uptake (Figure 2).

**Table 3:** Raw data from baseline to limit of tolerance during a maximal progressive graded exercise test.

Time	Speed	Elevation	VO_2_	VO_2_	VE	RER	HR	RPE
(min)	(km•h^−1^)	(%)	(mL•min^−1^)	(mL•kg^−1^•min^−1^)	(L•min^−1^)	(VCO_2_/VO_2_)	(s•min^−1^)	(BORG)
00:30	4.8	4	672	11.6	20.4	0.92	100	
01:00	4.8	4	755	13.1	18.9	0.92	121	
01:30	4.8	4	885	15.3	19.0	0.85	121	
02:00	4.8	4	1204	20.8	24.5	0.77	121	
02:30	4.8	4	1210	20.9	26.2	0.80	121	
03:00	4.8	4	1157	20.0	24.8	0.81	122	
03:30	4.8	4	1087	18.8	23.2	0.82	123	
04:00	4.8	6	966	16.7	20.4	0.80	123	
04:30	4.8	6	**1314^-1^**	**22.7^-1^**	27.9	0.83	124	8
05:00	4.8	8	**1227^-1^**	**21.2^-1^**	26.1	0.86	128	8
05:30	4.8	8	1319	22.8	29.9	0.91	135	8
06:00	4.8	10	1392	24.1	30.4	0.91	141	8
06:30	4.8	10	**1613^-2^**	**27.9^-2^**	37.0	0.95	143	8
07:00	4.8	12	**1582^-2^**	**27.4^-2^**	38.2	0.98	150	11
07:30	4.8	12	**1730^-3^**	**29.9^-3^**	42.0	0.98	150	11
08:00	4.8	14	**1608^-3^**	**27.8^-3^**	38.9	0.99	153	11
08:30	4.8	14	1775	30.7	41.8	0.97	155	11
09:00	4.8	16	**1848^-4^**	**32.0^-4^**	45.1	1.03	156	11
09:30	4.8	16	**1791^-4^**	**31.0^-4^**	43.1	1.07	160	11
10:00	4.8	18	**1656^-4^**	**28.6^-4^**	41.3	1.08	163	11
10:30	4.8	18	2065	35.7	54.8	1.12	164	12
11:00	4.8	20	2078	36.0	57.9	1.15	168	12
11:30	4.8	20	**2126^-5^**	**36.8^-5^**	63.7	1.19	171	12
12:00	5.3	20	**2010^-5^**	**34.8^-5^**	62.3	1.32	171	14
12:30	5.3	20	2122	36.7	69.2	1.27	175	14
13:00	5.8	20	**2259^-6^**	**39.1^-6^**	80.4	1.26	175	16
13:30	5.8	20	**2187^-6^**	**37.8^-6^**	90.2	1.25	177	18

HR  =  Heart rate; RER  =  Respiratory Exchange Ratio; RPE  =  Rating of Perceived Exertion; VE  =  Ventilation; VO_2_ =  Oxygen uptake.

The bold numbers followed by an elevated number indicate a plateau in oxygen uptake (VO_2_) during the test. Note that at least six plateau occurred after the 4. minute during the test, two after RER ≥1.15.

Based on the reasons mentioned above, and taking into account the fast electronic, real time gas analyzers that are available currently, the cutoff value proposed by Taylor seems to be too liberal, and should therefore not be used during continuous graded protocols, especially in elderly patients or in unfit subjects, for whom the increase in workload is low.

Reaching a plateau in VO_2_ during a progressive exercise test places great demands on the anaerobic energy consumption. This may be a challenge, especially for untrained or elderly subjects, who are not familiar with the unpleasant feelings associated with strenuous activities [26]. There was, however, no relationship between fitness level and age regarding achievement of a plateau in the current study, with the exception of the oldest age cohort of men (data not shown). Nevertheless, only 65% of those who reached a plateau fulfilled the blood lactate concentration criterion of ≥8.0 mmol•L^–1^.

#### Respiratory Exchange Ratio

In cases of failure to achieve a plateau in VO_2_, RER is the most-used secondary criterion for attaining VO_2max_ [8]. The rise in RER during heavy exercise is caused by an imbalance between the production and the elimination of lactic acid, because of the increase in the buffering of lactate [16]. In addition, as CO_2_ is generated from muscle work, the rise in ventilation increases the RER [8]. Therefore, it seems logical that if the blood lactate concentration is high, the RER would be high. This is in line with the results of the present study, which showed that 84% of the individuals with a blood lactate concentration ≥8.0 mmol•L^–1^, had RER ≥1.15.

Despite the fact that RER ≥1.15 is the originally recommended secondary end criterion [9], lower RER_max_ cutoff values have been used, such as ≥1.10 [6], ≥1.05 [3], or ≥1.0 [4]. Only 65% of the subjects enrolled in the present study reached RER ≥1.15. Even though the association with age was weak (r = –0.304), there was a reduction in RER in each age cohort after 50 years of age, despite the fact that the subjective ratings of perceived exertion was unchanged. The decrease in RER in the elderly is based on a shift from type II to type I fibers and corresponding metabolic shift towards an oxidative (lipid) preferential phenotype. Thus, RER should be adjusted for age when used as a criterion for establishing of VO_2max_.

#### Blood Lactate Accumulation

Blood lactate is a good indicator of a high effort, as high blood lactate levels are associated with fast-twitch fiber recruitment [27] and a progressive or sharp decrease in intracellular pO_2_ [28]. Here, the postexercise blood lactate concentration decreased with increasing age, especially after 50 years of age. Choosing the well-known 8 mmol·L^–1^ end criterion, which is based originally on findings from 14–18-year-old boys and girls [15], led to the exclusion of 63% of the participants in the 50–85-year-old cohort. Sidney and Shephard [29] also found a lower incidence of reaching a high level of blood lactate concentration in the elderly, even though a plateau in VO_2_ was achieved in these individuals. Lower blood lactate accumulation in the elderly may be explained by dyspnea, loss of type II fibers followed by muscular weakness, and lower capacity for anaerobic glycolysis [25]. Thus, this is an expected finding, and therefore the potential for lactate accumulation is reduced by age. Even though the RER value and the incidence of a plateau were similar between the sexes, women had a significantly lower blood lactate concentration compared with men. This is in accordance with previous studies performed using both trained and untrained subjects [30], [31] and suggests that men have a greater capacity than women to generate ATP via anaerobic glycolysis. In addition, women have a smaller ratio of muscle mass to total blood volume [29] and achieve lower workloads on the treadmill compared with men. A higher workload suggests greater energy turnover and more glycolytic flux, which may lead to greater lactate levels [32]. Such a difference between sexes should be taken into account when evaluating maximal effort using blood lactate as a criterion.

The assessment of postexercise blood lactate concentration is a non-manipulative variable, in contrast to RER (breathing pattern) or HR (psychological factors). This assessment is easy to perform and has a high measurement accuracy; thus, it represents a more objective physiological reflection of the amount of high-intensity exercise compared with VO_2_ leveling off, RER, or the percentage of the HR predicted. Despite the essential nature of this variable, we have only been able to find one epidemiological study reporting cardiorespiratory fitness variables in a population with measured blood lactate levels [33]. Based on the reasons mentioned above, we recommend that this variable should be used more frequently.

#### Maximal Heart Rate

HR_max_ differed significantly in all age cohorts from the commonly used formula of 220–age. Furthermore, the standard deviation was high (±15.0) in the 65–85-year-old cohort, making it very difficult to justify the use of this variable as a standard (because of its wide range). These findings are in line with those of previous studies [5], [24], and the use of a certain percentage of the age-adjusted HR_max_ has been questioned [8]. The American College of Sports Medicine stated over 20 years ago that age-predicted HR_max_ should not be used as an absolute criterion for maximal effort, which is supported by our data.

#### Rating of Perceived Exertion

The Borg Scale is widely used to measure exercise intensity, and there is a relationship between rating of perceived exertion and physiological measures such as HR and blood lactate concentration [34]. The Borg Scale has, however, produced inconsistencies regarding the strength of the relationships; in addition, its validity has been shown to be lower than was previously thought [35]. This is in agreement with the results of the current study, especially those of elderly men and women who scored high on the Borg Scale despite lower postexercise blood lactate concentration and RER.

### Choice of End Criteria and Impact on the VO_2max_ Value

Reaching objective variables of maximal effort has been shown to be difficult for athletes [19], [36], elderly people [13], [29], obese individuals [26], sedentary people, and patients [37], and depends on the measurement method used, sampling interval [38], and type and duration of the test protocol [20]. Furthermore, the standards used for each of the maximum criteria exhibited great variability. Some of these may be too low [5] or totally absent [39], which may increase the likelihood of underestimating the VO_2max_ variable; or the opposite, too high, leading to the rejection of subjects who would actually achieve a valid VO_2max_, thereby giving an overestimation of the VO_2max_. The choice of different end criteria for maximal effort in the present 20–85-year-old population had an impact on the number of participants included in each age cohort, sex, or the results, whereas a blood lactate concentration ≥6.0 and ≥8.0 mmol•L^–1^ and an RER ≥1.15 had the greatest impact on the VO_2max_ result.

Poole and co-workers [17] compared the VO_2max_ results obtained based on leveling-off criteria, which they defined as a true VO_2max_, with the RER, blood lactate concentration, and age-predicted HR_max_ in eight healthy young men performing a cycle ramp protocol until exhaustion. They found that terminating the exercise test immediately after reaching the RER criteria of 1.10 or 1.15 led to an underestimation of the VO_2max_ of as much as 27% and 16%, respectively, compared with the results obtained using leveling off. Furthermore, those authors found that the blood lactate concentration criterion was unusable because of the rejection of three out of eight participants (range, 5.7–8.4). However, the 8 mmol•L^–1^ blood lactate criterion is based on the use of hemolyzed blood, whereas Poole and co-workers used full blood (YSI 1500 Sport), which yields significantly lower results [40]. The two blood lactate techniques mentioned above are commonly used and should not be compared unless they have been corrected for the difference. In addition, while measuring gas exchange, most test laboratories currently use the breath-by-breath technique, rendering the interpretation of each measurement impossible during the test because of the considerable variability from measure to measure. Stopping the exercise test based on the measurement and not because of exhaustion or voluntary determination is thus not meaningful.

### New Recommendations

We have presented new recommendations for postexercise blood lactate concentrations and RER values (Table 4). These recommendations are based on the age and sex differences derived from the present results, as discussed previously, where both criteria must be fulfilled.

**Table 4:** New recommendations for maximal effort for haemolysed post exercise blood lactate* and respiratory exchange ratio (RER).

	Blood lactate concentration†	AND	RER
	(mmol•L^−1^)		(VCO_2_/VO_2_)
**Female**			
20–49 years	≥7.0		≥1.10
50–64 years	≥5.0		≥1.05
≥65 years	≥3.5		≥1.00
**Male**			
20–49 years	≥9.0		≥1.10
50–64 years	≥6.0		≥1.05
≥65 years	≥4.0		≥1.00

Measured one minutes after determination; †Does not equal values from full blood analysis; RER  =  Respiratory Exchange Ratio; VO_2_ =  Oxygen uptake; VCO_2_ =  Carbon dioxide output.

In our opinion, the use of VO_2_ leveling off is not recommended because of the achievement of several plateaus during a continuous graded exercise protocol, which is also supported by Noakes [41]. Therefore, it is easy to misinterpret these results during the test (Table 3, Figure 2). We chose the average value of blood lactate concentration and RER from each sex and age cohort minus one SD. This was chosen because the SD reflects the dispersion in each age cohort. However, using all accepted tests above one negative SD will reject 16% of the participants in each age group. As the concept of maximal oxygen uptake involves maximal aerobic energy metabolism, we have experienced that many will struggle to push enough to reach the needed level of exhaustion.

### Strengths and Limitations

The main strengths of the current study were its large sample of both fit and unfit men and women, the random inclusion of participants from rural and nonrural populations, and the wide age range of the participants.

One of the limitations of the study was the use of nine different test laboratories, including a large number of technicians, which may have increased the possibility of different levels of encouragement regarding maximal effort, in addition to the possibility of achieving different measurement accuracies across laboratories. However, some initiatives were taken to minimize these issues. First, all the technicians were rigorously trained in all test procedures and they were experienced with maximal-exercise testing. Second, all gas analyzers were checked for measurement precision and accuracy using a standardized motorized mechanical lung (Motorized Syringe with Metabolic Calibration Kit; VacuMed, Ventura, CA, USA). Third, use of different technicians reflects the “real life” situation, thus being more representative.

## Conclusion

A range of typical end criteria were presented in a random sample of healthy men and women aged 20–85 years. The choice of end criteria during exercise testing had an impact on sex and the number of participants, and some impact on the outcome of the test. Based on these results, new recommendations are given according to age and sex for individuals using a continuous graded exercise test on a treadmill. Studies with other populations should be applied to confirm our results.
